# Effect of reconstruction methods and x-ray tube current–time product on nodule detection in an anthropomorphic thorax phantom: A crossed-modality JAFROC observer study

**DOI:** 10.1118/1.4941017

**Published:** 2016-02-11

**Authors:** J. D. Thompson, D. P. Chakraborty, K. Szczepura, A. K. Tootell, I. Vamvakas, D. J. Manning, P. Hogg

**Affiliations:** Directorate of Radiography, University of Salford, Frederick Road Campus, Salford, Greater Manchester M6 6PU, United Kingdom and Department of Radiology, Furness General Hospital, University Hospitals of Morecambe Bay NHS Foundation Trust, Dalton Lane, Barrow-in-Furness LA14 4LF, United Kingdom; Department of Radiology, University of Pittsburgh, FARP Building, Room 212, 3362 Fifth Avenue, Pittsburgh, Pennsylvania 15213; Directorate of Radiography, University of Salford, Frederick Road Campus, Salford, Greater Manchester M6 6PU, United Kingdom; Department of Radiology, Christie Hospitals NHS Foundation Trust, 550 Wilmslow Road, Manchester M20 4BX, United Kingdom; Faculty of Health and Medicine, Lancaster Medical School, Furness College, Lancaster University, Lancaster LA1 4YG, United Kingdom; Directorate of Radiography, University of Salford, Frederick Road Campus, Salford, Greater Manchester M6 6PU, United Kingdom and Department of Radiography, Karolinksa Institute, Solnavägen 1, Solna 171 77, Sweden

**Keywords:** CT, Iterative reconstruction, JAFROC, CNR, effective risk

## Abstract

**Purpose:**

To evaluate nodule detection in an anthropomorphic chest phantom in computed tomography (CT) images reconstructed with adaptive iterative dose reduction 3D (AIDR^3D^) and filtered back projection (FBP) over a range of tube current–time product (mAs).

**Methods:**

Two phantoms were used in this study: (i) an anthropomorphic chest phantom was loaded with spherical simulated nodules of 5, 8, 10, and 12 mm in diameter and +100, −630, and −800 Hounsfield units electron density; this would generate CT images for the observer study; (ii) a whole-body dosimetry verification phantom was used to ultimately estimate effective dose and risk according to the model of the BEIR VII committee. Both phantoms were scanned over a mAs range (10, 20, 30, and 40), while all other acquisition parameters remained constant. Images were reconstructed with both AIDR^3D^ and FBP. For the observer study, 34 normal cases (no nodules) and 34 abnormal cases (containing 1–3 nodules, mean 1.35 ± 0.54) were chosen. Eleven observers evaluated images from all mAs and reconstruction methods under the free-response paradigm. A crossed-modality jackknife alternative free-response operating characteristic (JAFROC) analysis method was developed for data analysis, averaging data over the two factors influencing nodule detection in this study: mAs and image reconstruction (AIDR^3D^ or FBP). A Bonferroni correction was applied and the threshold for declaring significance was set at 0.025 to maintain the overall probability of Type I error at *α* = 0.05. Contrast-to-noise (CNR) was also measured for all nodules and evaluated by a linear least squares analysis.

**Results:**

For random-reader fixed-case crossed-modality JAFROC analysis, there was no significant difference in nodule detection between AIDR^3D^ and FBP when data were averaged over mAs [*F*(1, 10) = 0.08, *p* = 0.789]. However, when data were averaged over reconstruction methods, a significant difference was seen between multiple pairs of mAs settings [*F*(3, 30) = 15.96, *p* < 0.001]. Measurements of effective dose and effective risk showed the expected linear dependence on mAs. Nodule CNR was statistically higher for simulated nodules on images reconstructed with AIDR^3D^ (*p* < 0.001).

**Conclusions:**

No significant difference in nodule detection performance was demonstrated between images reconstructed with FBP and AIDR^3D^. mAs was found to influence nodule detection, though further work is required for dose optimization.

## 1. INTRODUCTION

Radiation dose in computed tomography (CT) is a highly topical concern in medical imaging and there is a recognition of increased dose with the use of multidetector CT (MDCT).^1–5^ However, radiation dose risk needs to be balanced with benefits, and MDCT has been a significant development in acute medicine^6–9^ where a quick and accurate diagnosis is important for patient outcome.

Low noise and high spatial resolutions are important considerations for accurate radiological CT reports. Until recently, filtered back projection (FBP) had been the image reconstruction method of choice. Unfortunately, data nonlinearity and image reconstruction artifacts are prevalent with FBP and a loss of spatial resolution is an unwanted trade-off when attempting to reduce image noise.^10–12^ Improved computer processing capability currently allows the use of iterative reconstruction (IR) in CT as an alternative to FBP. Incorporating physical models into the algorithm allows image quality to be maintained at lower dose and lower noise levels,^13^ and dose reductions ∼23%–79% have been reported when using IR in place of FBP.^14–17^

Adaptive iterative dose reduction 3D (AIDR^3D^, Toshiba Medical Systems, Minato-ku, Japan) is a recently developed IR algorithm for CT data, where it has been suggested that using AIDR^3D^ in place of FBP could allow dose saving ∼75%.^18^ A detailed explanation of how AIDR^3D^ works in the projection and reconstruction domains has been published.^19^

Several studies^19–21^ have assessed this new algorithm using objective and subjective measures. Common to all is an objective evaluation of image noise, evaluating either the standard deviation of pixel values in regions of interest in various quasiuniform anatomical regions^19,20^ or the noise power spectrum (NPS) in a phantom model.^21^ All studies report reductions in image noise. Spatial resolution was assessed objectively using the modulation transfer function^21^ and subjectively using a five-point scale to assess the pulmonary vessels;^20^ both of these methods suggested that spatial resolution (or sharpness) was reduced with AIDR^3D^ in comparison to FBP.^20,21^ Subjective evaluations of images, using five-point visual scoring systems, were used to assess diagnostic acceptability,^19–21^ artifacts,^20,21^ and pathology.^19^ With one exception,^20^ subjective image quality was stated as being better with AIDR^3D^. Despite the inconsistencies listed above, all studies suggested that AIDR^3D^ could offer a large dose reduction in the thorax by a factor of 6 from 150 down to 25 mAs,^19^ when using a low dose acquisition in place of a standard dose acquisition,^20^ or as an average of 36% over a range of mAs settings (comparing FBP and AIDR^3D^ directly).^21^ Ohno *et al.*^19^ and Yamada *et al.*^20^ used the computed tomography dose index (CTDI) to assess radiation dose, when in fact it is only a measure of absorbed dose to a standardized phantom and does not account for patient size and potential cancer risk.^22^ The above studies are further limited by a the lack of a ROC type analysis and low case numbers, e.g., 37 (Ref. 19) and 50 (Ref. 20) patients, respectively.

We are aware of only one paper that assesses the value of IR in the thorax with observer performance methods. A study by Katsura *et al.*^23^ assessed the value of using a model-based IR algorithm (MBIR) against adaptive statistical IR (ASIR; GE Healthcare, Waukesha, WI). An ultralow-dose MBIR acquisition with a fixed tube current was compared to a low-dose acquisitions using ASIR and automatic tube current modulation. The study used 59 patients and 2 observers, with 84 nodules present in 41 patients, with the remaining 18 patients having no lung nodules. Nodule detection rates were similar between the two acquisitions (*p* = 0.57), and the authors reported dose saving of more than a factor of 4, from a DLP of 66 to 14.5 mGy cm. However, it is not possible to claim that nodule detection rates were equivalent without performing an equivalence study.^24,25^ Stated simply, not being able to reject the null hypothesis of equal performance does not imply that the two modalities have equal performances. The work of Katsura *et al.* differs from previous work and this study in that they compared two IR algorithms and not FBP. In this work, we make methodological improvements on previous studies to evaluate the performance of AIDR^3D^ and FBP for nodule detection over a range of mAs. Initial results questioning the advantages of IR over FBP in an anthropomorphic chest phantom were presented as a conference paper in early 2015.^26^

## 2. METHOD

A free-response study was conducted using an anthropomorphic chest phantom to determine nodule detection performance for images constructed using FBP and IR over a range of mAs values. This was combined with an accurate assessment of radiation dose using a separate phantom.

### 2.A. Phantoms

An anthropomorphic chest phantom (Lungman N1 Multipurpose Chest Phantom, Kyoto Kagaku Company, Japan) representing a 70 kg male was loaded with simulated nodules measuring 5, 8, 10, and 12 mm in spherical diameter and +100, −630, and −800 Hounsfield unit (HU) densities. The higher electron density (+100 HU) nodules are composed of polyurethane, hydroxyapatite, and a urethane resin; the lower electron density (−630 and −800 HU) nodules are composed of urethane.

An ATOM 701D (ATOM 701; CIRS, Norfolk, VA) whole-body dosimetry verification phantom was used to measure organ doses. Prior to data collection, the median-sagittal and midcoronal planes, and the scan range that covered the lung apices and costodiaphragmatic recesses, were marked on the dosimetry phantom using an indelible marker. This allowed accurate and reproducible positioning and scanning of the dosimetry phantom.

### 2.B. Image acquisitions

**FIG. 1.**
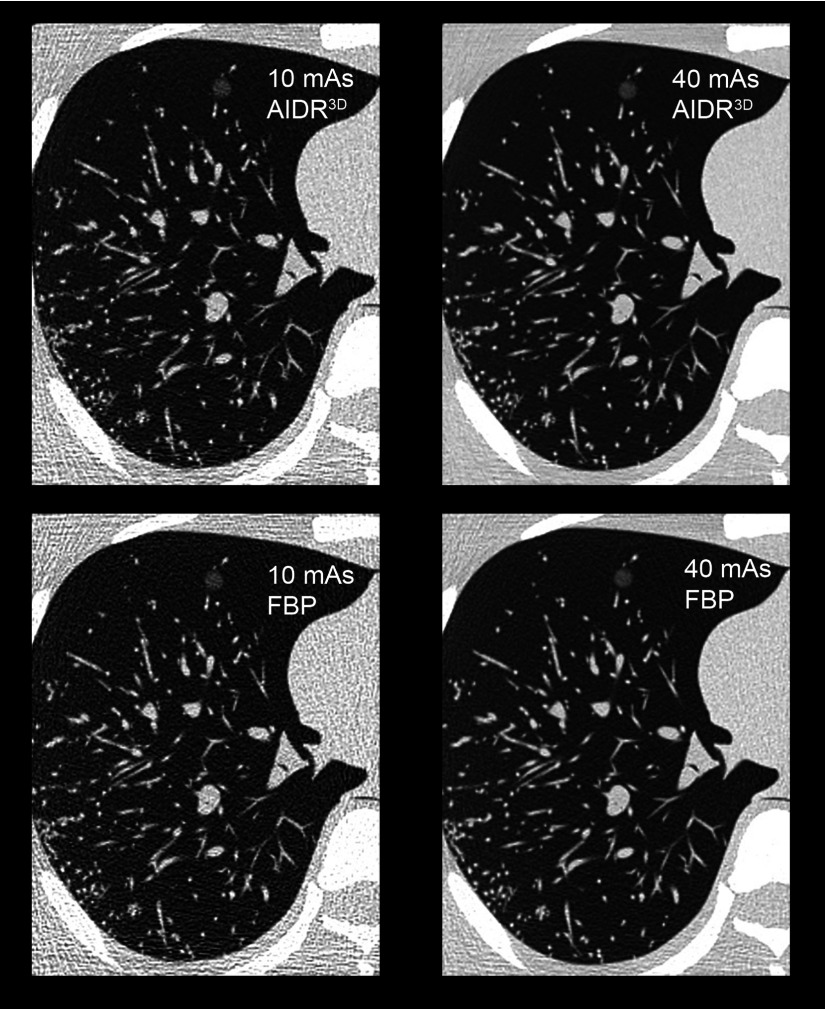
Sample images to demonstrate the effect of mAs (horizontally) and reconstruction method (vertically). A simulated nodule measuring 10 mm and −630 HU is visualized in the anteromedial aspect of the simulated lung.

All image acquisitions were completed on a Toshiba Aquilion One 320-slice MDCT scanner (Toshiba Medical Systems,Minato-ku, Japan) in volume mode. Each volume covered 160 mm in the transaxial (*z*-axis) plane, where the volume is also the collimation size in this instance, and three volumes were required to provide complete coverage of the anthropomorphic chest phantom. A mAs range (10, 20, 30 and 40 mAs) was investigated for both reconstruction algorithms (FBP and IR), while all other CT acquisition parameters remained constant (120 kVp, 0.5 s rotation time, pitch 1, 64 × 0.5 mm detector configuration, 1 mm slice reconstruction, 512 × 512 matrix size, 320 mm scan and reconstruction field of view, and 0.625 mm pixel size, a medium bowtie filter, appropriate for the 320 mm field of view). The images were reconstructed with FBP and AIDR^3D^, Fig. 1.

The anthropomorphic chest phantom was loaded with three different nodule configurations. Nodules were distributed as described in Table I, with nodules considered peripheral if they were in close proximity to the chest wall. For each mAs and image reconstruction, 34 abnormal transaxial image slices (containing 1–3 nodules, mean 1.35 ± 0.54) and 34 normal transaxial image slices corresponding to the same anatomical position for each modality were chosen for the observer study. Nodule positions were recorded at the time of insertion and confirmed on the lowest noise images (40 mAs, reconstructed with AIDR^3D^) to act as the truth (gold standard) for the observer study.

### 2.C. Dosimetry

TLDs (TLD100H LiF:Mg,Cu,P, Thermo Scientific, Waltham, MA) (*n* = 271 plus *n* = 5 for background correction) were grouped into batches of similar response (intrabatch variation ≤ 2%). Processing of the TLDs was carried out using Harshaw 3500 manual TLD reader (Thermo Scientific, Waltham, MA). Each batch of TLDs was calibrated. Annealed TLDs were positioned within the dosimetry phantom at locations corresponding to 23 of the critical organs identified in ICRP report 103 (Ref. 27) for each of the four imaging conditions. Effective dose was calculated from the organ doses by applying radiation and tissue-weighting factors specified in the same publication.^27^

Effective risk was calculated using pcxmc software (STUK, Helsinki, Finland), a Monte Carlo program for estimating patient doses. The software estimates the patient risk of death due to radiation-induced cancer, according to the risk model of the BEIR VII committee.^28,29^

This CT system acquires data in volume mode. The volume of 160 mm is not fully contained within the dimensions of a typical CT dose phantom and standard pencil CT ionization chamber.^30^ This CT scanner reports CTDI_vol_ values that are adjusted for wide beam CT when acquiring data in volume mode.

### 2.D. Observer study

Six radiologists (12.2 ± 9.1 yr reporting experience) and five radiographers were trained to perform CT examination (18 ± 5.3 yr CT imaging experience) who completed the observer study. For each combination of mAs and reconstruction method, each observer interpreted 68 cases (i.e., single transaxial CT images) using the free-response receiver operating characteristic (FROC) paradigm. The interpretations were performed in two sessions, each lasting approximately 1 h. Each observer viewed the cases in a different randomized order. They were unaware of the mAs and reconstruction methods used to generate each image, but were informed that half of the images contained 1–3 simulated nodules of varying sizes and contrasts and the remaining contained none. All observers completed a training exercise prior to the main study. Ten nonidentifiable images containing nodules and ten not containing any nodules, which were cases not used in the main study, were used to demonstrate the appearance of the anthropomorphic chest phantom and simulated nodules, while also giving the opportunity to learn how to localize nodules and use the rating scale and familiarize themselves with the user interface. The same monitor (PG21HQX, Wide, 20 in., LCD, Wide Corporation, Korea) (1536 × 2080 pixels, 3.2 megapixel resolution) was used for all observers and evaluations under the same controlled viewing conditions.

The FROC method was used to acquire the observer data. Observers were instructed to mark the center of each simulated nodule using a single mouse click; this would cause a “pop-up” a slider bar rating scale to appear by which they could rate confidence on a 1–10 integer scale. Using a 20-pixels acceptance radius, marks were classified as nodule localization (LL) if they were within the acceptance radius of the nearest nodule or non-nodule localization (NL). Image display and FROC study functionality were managed by ROCView (Ref. 31) display and data acquisition software. Images were viewed on a fixed lung window (1500, −500) to maximize nodule visibility and reduce observer variability.

### 2.E. Statistical analysis

In this study, the equally weighted jackknife alternative FROC (JAFROC) figure of merit was used, denoted by *θ*.^32^ The JAFROC figure of merit is the weighted empirical probability that a nodule rating is higher than any rating on a normal case.^32^ In this study, all nodules on a case were assigned the same weight. The weighting gives equal importance to each case, independent of the number of true nodules in it. To check for consistency, inferred-ROC analysis was also performed. To do this, we used the highest rating on a case to define the inferred-ROC rating for that case.

In this study, there were two *factors* (in the statistical sense) that would ultimately influence the performance of the observer - mAs and image reconstruction method. In a typical analysis of multimodality multiple reader multiple case, typically termed as a MRMC ROC/FROC study, modality is considered as a single factor with *I* levels, where *I* is usually small, but greater than 2. For example, if comparing two image reconstruction methods, *I* = 2. The measure of performance or figure of merit for modality $i\left(i=1,2,\ldots ,I\right)$ and reader *j*(*j* = 1, 2, …, *J*), where *J* is the number of readers, is denoted as *θ_ij_*. Current MRMC ROC/FROC analysis compares the observed difference in reader-averaged figures of merit between modalities *i* and $i^{\prime }\;\left(i\neq i^{\prime }\right)$ to the estimated variability of the difference. For example, the reader-averaged difference in figures of merit is *θ*_*i*⋅_ − *θ*_*i*′⋅_, where the dot symbol represents the average of the corresponding index, specifically, the reader index. The variability of the difference is estimated using the Hillis-modified Obuchowski–Rockette (ORH) method,^33^ with resampling (i.e., jackknifing) used to determine the two covariances needed for the ORH method. With *I* levels, the number of possible *i* versus *i*′ comparisons is *I*(*I* − 1)/2. If the current study were analyzed in this manner, where *I* = 8 (4 levels of mAs and two image reconstruction methods), then this would imply 28 comparisons. The large number of comparisons is suboptimal in terms of statistical power and does not inform us of the main points of interest: whether performance depends on (i) mAs and/or (ii) reconstruction method.

Unlike conventional ROC type studies, the images in this study are defined by two factors. The first factor, mAs, had four levels: 10, 20, 30, and 40 mAs. The second factor, reconstruction method, had two levels: FBP and AIDR^3D^. Each factor is combined with the other, so they are *fully crossed factors* (in the statistical sense). The figure of merit is represented by *θ*_*i*_1_*i*_2_*j*_, where $i_{1}\left(i_{1}=1,2,\ldots ,I_{1}\right)$ represents the levels of the first factor (mAs), *I*_1_ = 4, and $i_{2}\left(i_{2}=1,2,\ldots ,I_{2}\right)$ represents the levels of the second factor (reconstruction method), *I*_2_ = 2. This called for two sequential analyses to be performed: the first was *mAs analysis*, where the figure of merit was averaged over the *i*_2_ or the reconstruction index; the second was *reconstruction analysis*, where the figure of merit was averaged over the *i*_1_ or the mAs index. For example, the *mAs analysis* figure of merit difference is *θ*_*i*_1_⋅⋅_ − *θ*_*i*′⋅⋅_, where the first dot represents the average over the *reconstruction* index and the second dot represents an average over readers. In either analyses, the figure of merit is dependent on only a single factor, and therefore, the standard ORH method applies.

The *mAs analysis* determines whether there is a mAs effect and in this analysis, the number of possible comparisons is six. The *reconstruction analysis* determines whether AIDR^3D^ offers any advantage over FBP and in this analysis, the number of possible comparisons is one. Multiple testing on the same dataset increases the probability of Type I error; therefore, a Bonferroni correction (Appendix A) was applied by setting the threshold for declaring significance at 0.025; this is expected to conservatively maintain the overall probability of a Type I error at *α* = 0.05. We use the term *crossed-modality* analysis to describe this type of analysis of ROC/FROC data.

Since the phantom is unique, and conclusions are only possible that are specific to this one phantom, the case (or image) factor was regarded as fixed. For this reason, only results of random-reader fixed-case analyses are reported. Software for *crossed-modality* modified JAFROC analysis was implemented in the r programming language^34^ and is downloadable from the https://cran.r-project.org/web/packages/RJafroc/index.html.

A Welch’s independent sample *t*-test was performed to assess any difference in performance between radiologists (*n* = 6) and radiographers (*n* = 5); the null hypothesis of no difference was tested at an alpha of 0.05.

### 2.F. Contrast-to-noise (CNR) ratio of nodules

The CNR of all nodules was measured using ImageJ (Ref. 35) software. The CNR is a measure of image quality based on contrast (in this instance between nodule and background), rather than the raw signal.^36^ Nodule measurements were made on images viewed by the observer. A region of interest (ROI) was placed just within the outer edge of each nodule and the mean pixel value was recorded. A background ROI was placed within a portion of the lung field containing no nodule or vascular markings, and the mean pixel value and standard deviation were recorded. A linear least squares analysis was performed to determine the impact of mAs and image reconstruction method on the CNR of all nodules. Test alpha was set at 0.05 for detecting significant differences in CNR between images reconstructed with FBP and AIDR^3D^.

## 3. RESULTS

A Welch’s unpaired *t*-test of observer averaged figures of merit revealed no significant difference in nodule detection performance between radiologists and CT trained radiographers (*p* = 0.1124, mean difference 0.051 [95% CI (−0.015,0.117)]). Based on this, all observers were included in the subsequent analysis.

**FIG. 2.**
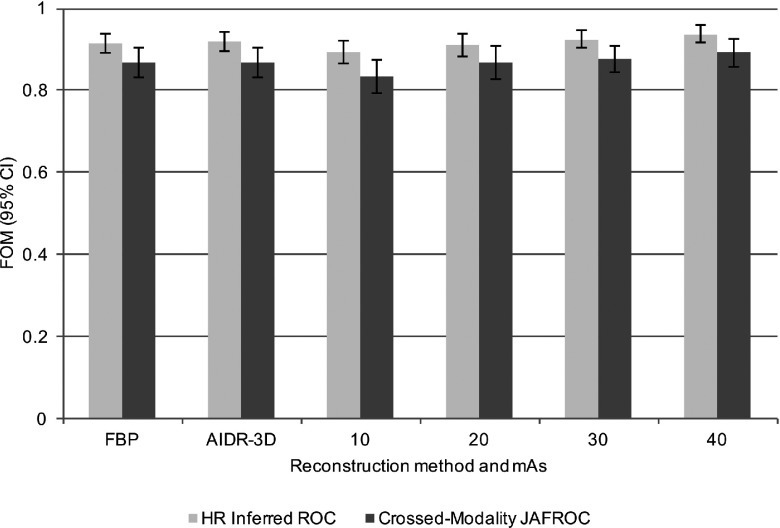
Figures of merit and 95% CI for crossed-modality JAFROC analysis (top) and highest-rating inferred ROC analysis (bottom).

**FIG. 3.**
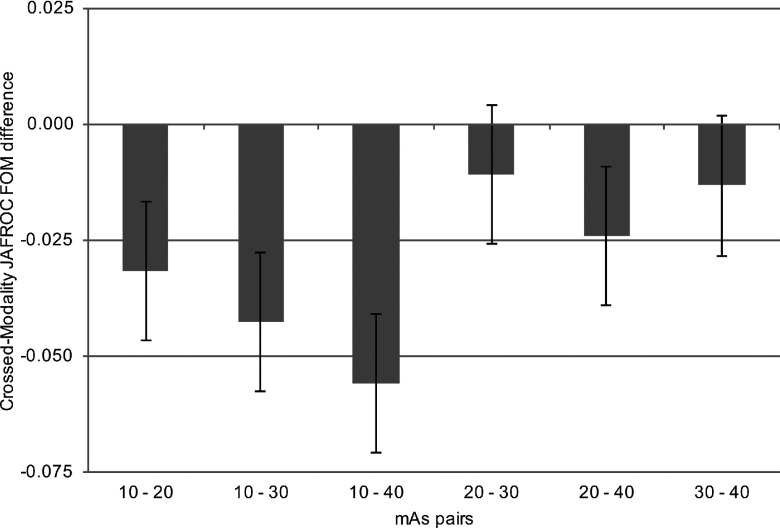
Intertreatment differences for crossed-modality modified JAFROC analysis. A difference is considered significant if the 95% confidence interval does not include zero and the *p*-value of the overall *F* test is less than 0.025. Statistical differences are seen between 10 and 20, 10 and 30, 10 and 40, and 20 and 40 mAs.

**FIG. 4.**
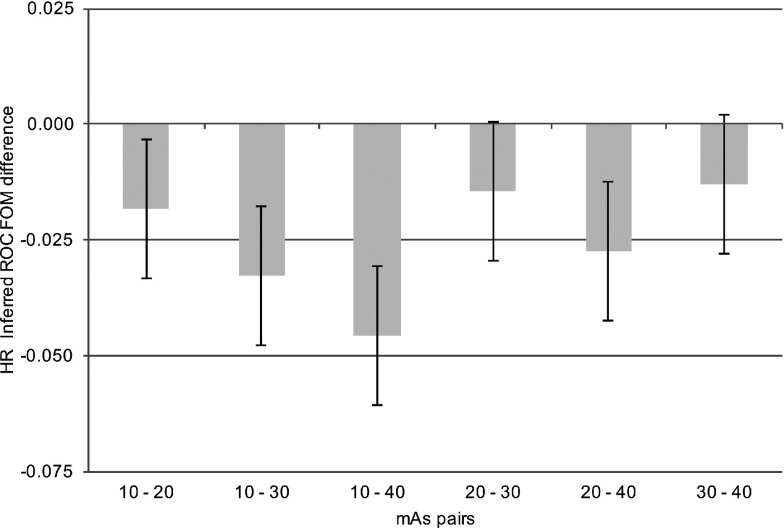
Intertreatment differences for the inferred ROC analysis. Statistical differences are observed for the same treatment pairs, as with JAFROC FOM in Fig. 3, with slight variation in the magnitudes of the differences (as expected, the inferred ROC differences are smaller in magnitude than the corresponding JAFROC FOM differences).

For a statistically significant difference to be declared, the *p*-value of the treatment pair *t*-test and that of the overall *F*-test must both be significant (Appendix B). For the first of the sequential *crossed-modality* JAFROC analyses, the *mAs analysis*, where the figure of merit is averaged over the *i*_2_ or the reconstruction index, significant differences were revealed between multiple pairs of mAs settings [*F*(3, 30) = 15.96, *p* < 0.001]. For the second of the sequential analyses, the reconstruction analysis, where the figure of merit was averaged over the *i*_1_ or the mAs index, there was no statistically significant difference in nodule detection performance between FBP and AIDR^3D^ [*F*(1, 10) = 0.08, *p* = 0.789]. Individual figures of merit are displayed in Table II and Fig. 2; intertreatment differences are presented in Fig. 3. The intertreatment differences for inferred ROC analysis are presented in Fig. 4. These yielded similar results; *mAs analysis* [*F*(3, 30) = 15.18, *p* < 0.001] and *reconstruction analysis* [*F*(1, 10) = 0.27, *p* = 0.615], i.e., consistent with *crossed-modality* JAFROC analysis. The important outcome is that no statistical difference was demonstrated between images reconstructed with FBP and AIDR^3D^. A statistically strong effect (*p* < 0.001) was seen with mAs. Figure 3 shows weighted JAFROC FOM differences and 95% confidence intervals (CIs) for all six pairings of mAs. A difference is significantly different from zero if the corresponding confidence interval does not include zero. Figure 3 shows that except for the 20–30 mAs and 30–40 mAs comparisons, the rest were all statistically significant. Figure 4 shows corresponding results using the inferred ROC FOM: the results are consistent with those shown in Fig. 3. As expected, the inferred ROC differences are smaller in magnitude than the corresponding JAFROC FOM differences (the ROC FOM ranges from 0.5 to 1, while the JAFROC FOM ranges from 0 to 1).

Statistically significant differences in nodule detection performance were observed between multiple pairs of mAs settings when the *p*-value of the overall *F*-test was *p* < 0.001; significant pairs were 10 and 20 mAs (*p* < 0.001), 10 and 30 mAs (*p* < 0.001), 10 and 40 mAs (*p* < 0.001), and 10 and 20 mAs (*p* = 0.008); no difference was found between 20 and 30 mAs or between 30 and 40 mAs (*p* > 0.025).

The results of effective dose and effective risk are summarized in Table III. The observations are consistent with the expected strict linear dependence of dose on mAs. The CTDI_vol_ values for each mAs setting are also reported.

**FIG. 5.**
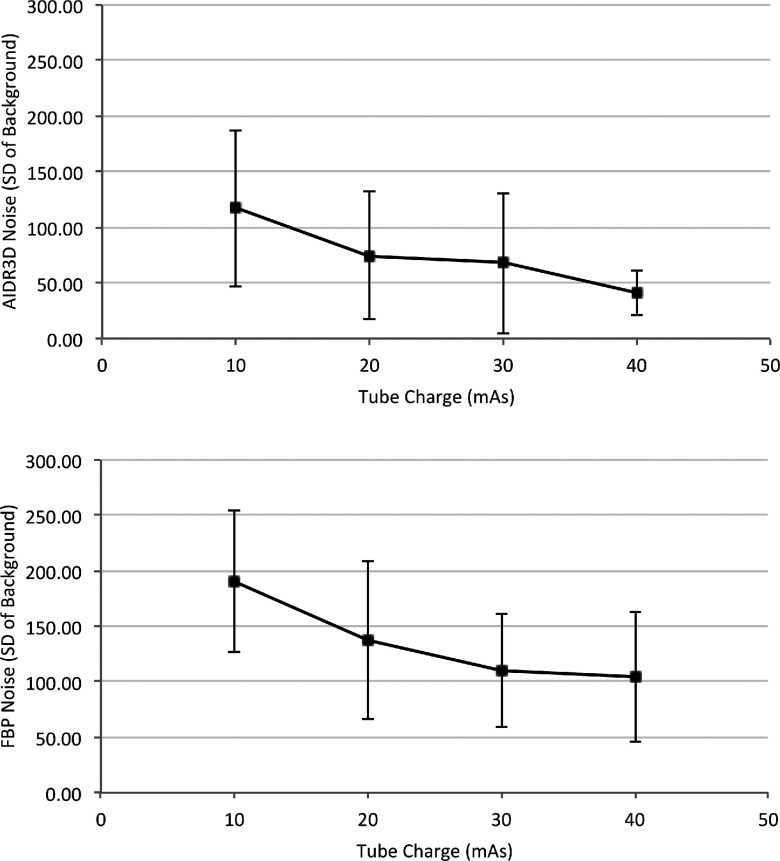
The mean noise and standard deviation as calculated from all images containing nodules for AIDR^3D^ (top) and FBP (bottom), respectively, over the mAs range investigated in this study. The difference in image noise between reconstruction methods was significant (*p* < 0.001) according to a least squares analysis.

Results for CNR are summarized in Table IV. Analysis by least squares revealed that measures of CNR were statistically higher for the simulated nodules on images reconstructed with AIDR^3D^ (*p* < 0.001). The reconstruction method did not impact on the contrast between nodule and background (*p* = 0.223), but the image noise was statistically higher on images reconstructed with FBP (*p* < 0.001). This is to be expected as the HU of the nodules and background should not change when using different reconstruction methods, and therefore, the only variable element within the CNR is the image noise. The relationship between image noise and mAs for each image reconstruction method is demonstrated in Fig. 5. Mean noise is lower at all mAs settings for images reconstructed with AIDR^3D^ in place of FBP. At 40 mAs, the noise level is very consistent when images are reconstructed with AIDR^3D^ and demonstrated by the small standard deviation.

## 4. DISCUSSION

This study has evaluated nodule detection in CT images reconstructed with AIDR^3D^ and FBP over a range of mAs. We found no statistically significant difference in nodule detection when images were reconstructed with either FBP or AIDR^3D^. However, we did find that the level of image noise was statistically higher in images reconstructed with FBP. This disparity, consistent with earlier studies, between image noise, a physical measure, and nodule detection, an observer performance measure, is an important finding given the steps taken to improve statistical power in this study. We removed case variability through the use of a phantom and the large number of readers (*n* = 11) used minimized reader variability, the *crossed-modality* methodology averaged the data over all mAs settings for a more stable measure and taking location into account, i.e., FROC study, increases statistical power compared to the ROC method. The other important finding of this work, evident in Fig. 2, is that mAs was found to have a significant effect on nodule detection, with detection compromised below 20 mAs as compared to 40 mAs. However, the fact that the 95% CI for the 20–30 comparison, includes zero, does not imply that the two are equivalent. A different type of statistical procedure is needed to infer equivalence between the two mAs settings.^24^ Software for this type of testing is not readily available.

Many previous studies^19–21,37,38^ have found a similar result to the present study when assessing image noise, be it by measuring CNR, NPS, or signal-to-noise ratio (SNR): they all find that the physical metrics improve as a result of reduced image noise with the IR algorithm. Our study is consistent with previous results: significant effect on physical measures between processing algorithms but insignificant effect in objective observer performance. We believe that the difference is due to the fact that an objective FROC observer performance measure, such as used in this study, takes the combined effect of all factors affecting nodule detectability into account, including visual search, while physical measure focuses on a few individual measures in isolation and does not account for visual search. Moreover, the physical measures considered in this paper do not represent state-of-the-art because they do not account for spatial corrections in the images. Newer model observer methods account for some of these correlations,^39–41^ and they are just beginning to account for visual search.^42^ However, observer performance studies suffer from much larger sources of variability than physical measures, so more careful statistical analysis is needed. As noted by the late Dr. Robert F. Wagner, finding physical measures, or combinations of physical measures, that correlate with the more time-consuming observer measures is one of the “holy grails” of medical imaging.^43^

Diagnostic acceptability must be maintained when looking to optimize the dose delivered to the patient. Many studies have suggested that IR algorithms can be used to optimize dose with a range of percentage reductions previously quoted (36%–75%).^18–20^ This requires the preoptimization start point to be reasonable and it is the postoptimization dose that should be given the greatest consideration.

For true optimization, the risk to the patient must also be understood. Patient dose is frequently reported using suboptimal estimation methods (CTDI, DLP, body part specific conversion factors) and the lifetime risk associated with x-ray exposures is rarely reported. The method used in the current work is considered a reliable method to accurately represent dose and risk and we would encourage future studies of IR algorithms to adopt this technique.

Lee *et al.*^44^ quote a mean effective dose of 1.84 ± 1.05 mSv in a study of pediatrics, where the purpose of the examination was to evaluate lung metastases. The mean weight of the patients was 41.4 kg, somewhat lighter than the estimated 70 kg patient size in our study. When using ASIR–FBP blending and FBP alone, Qi *et al.*^45^ showed that radiation dose to the patient could be optimized at an average effective dose of 4.25 mSv (range 2.6–6.3 mSv) with ASIR–FBP blending compared to an average of 8.65 mSv (range 7.9–9.5 mSv) with FBP alone. This finding is supported by Chen *et al.*^46^ but their postoptimization dose was much lower at 0.74 mSv. Both studies investigated ASIR in a patient population, and both proposed ASIR blending at 50% as being optimal. The large difference in estimated effective dose in these studies is likely due to the amount of noise permitted in the images by the automatic exposure control (AEC) and image quality paradigm. The noise index, in the GE systems of the above papers, is referenced to the standard deviation of pixel values in a water phantom, compared to patient attenuation measured in the CT planning image, in order to maintain a constant level of image noise.^47^ Qi *et al.* chose a noise index of 15, while Chen *et al.* chose a noise index of 30, where a higher noise index provides a greater reduction in tube current. Neither observer performance evaluation nor equivalency study was performed in either of these works, and further assessment is required before dose optimization can be claimed with IR algorithms.

Previous optimistic claims of dose reduction with IR algorithms are mainly based on physical measures. While our methods were sensitive enough to find statistical differences in nodule detection performance attributed to mAs, we were unable to detect any statistical difference in nodule detection on the basis of image reconstruction algorithm. It is not surprising that pixel-variance is a poor predictor of lesion detectability; for example, it can be reduced almost arbitrary, by smoothing the image. The inadequacy of pixel variance as a predictor of lesion detectability was noted in 1999 by Burgess, but this work is not well appreciated.^48^ IR algorithms require further investigation, with observer performance and equivalency testing playing a more prominent role.

## 5. CONCLUSION

We have successfully demonstrated the use of a *crossed-modality* JAFROC analysis that allows us to take coexisting factors into account in order to determine the dependence of nodule detection on each factor. We believe that this is a useful methodological improvement, since system performance is usually dependent on more than just a single factor. No significant difference in nodule detection performance was demonstrated between images reconstructed with FBP and AIDR^3D^. mAs was found to influence nodule detection, but further work is required for dose optimization.

## Figures and Tables

**TABLE I.** The spatial distribution of nodules (*n* = 46) used in the observer study. The nodules were distributed 25:21 (right:left); 8:26:12 (upper:mid:lower); 8:25:13 (anterior:posterior:central). Nodules were considered to be peripheral (*n* = 18) if they were in close proximity (2 cm) to the chest wall.

	Anterior		Posterior		Central		Peripheral	
Zone	Right	Left	Right	Left	Right	Left	Right	Left
Upper	—	3	3	1	—	1	—	2
Mid	2	1	9	7	3	4	2	5
Lower	2	—	3	2	3	2	4	5

**TABLE II.** Figures of merit and 95% CIs for crossed-modality JAFROC analysis and highest-rating inferred ROC analysis. Analysis *i*_1_ is averaged over mAs and analysis *i*_2_ is averaged over reconstruction method (FBP or AIDR^3D^).

Averaged over index	Reconstruction method and mAs	Crossed-modality JAFROC FOM (95% CI)	Inferred ROC FOM (95% CI)
*i*_1_	FBP	0.867 (0.832,0.903)	0.918 (0.894,0.941)
	AIDR^3D^	0.869 (0.833,0.904)	0.915 (0.891,0.938)
*i*_2_	10 mAs	0.835 (0.769,0.875)	0.892 (0.865,0.919)
	20 mAs	0.867 (0.827,0.908)	0.910 (0.884,0.937)
	30 mAs	0.878 (0.864,0.910)	0.925 (0.904,0.945)
	40 mAs	0.891 (0.858,0.925)	0.938 (0.916,0.960)

**TABLE III.** Effective dose and effective risk for the mAs range investigated in this study. An approximately linear increase in dose and risk is observed.

mAs	10	20	30	40
CTDI_vol_ (mGy cm^2^)	0.7	1.4	2.1	2.6
Effective dose (mSv)	0.49	0.97	1.64	2.11
Effective risk (%)	1 in 714 (0.0014)	1 in 345 (0.0029)	1 in 208 (0.0048)	1 in 167 (0.0060)

**TABLE IV.** A summary of the mean contrast-to-noise ratio of all simulated nodules, mean contrast between nodule and background, and the mean noise levels on all images containing nodules for both reconstruction methods and over the range of mAs investigated in this study.

		Mean values and standard deviation			
mAs		10	20	30	40
Nodule CNR	FBP	2.58 ± 2.42	3.36 ± 3.43	4.13 ± 4.46	4.90 ± 4.65
	AIDR^3D^	4.52 ± 4.30	6.85 ± 7.15	9.17 ± 9.27	11.5 ± 9.16
Contrast	FBP	423 ± 316	412 ± 324	443 ± 337	419 ± 407
	AIDR^3D^	410 ± 315	471 ± 318	441 ± 336	417 ± 311
Noise	FBP	190 ± 63.6	137.53 ± 71.1	110 ± 51.41	104 ± 58.5
	AIDR^3D^	117 ± 70.0	74.7 ± 57.4	67.6 ± 63.4	40.9 ± 19.7

## APPENDIX A: BONFERRONI CORRECTION

Under null hypothesis (NH) true conditions, a valid significance testing procedure maintains the probability of a Type I error (incorrect rejection of the NH) at the chosen value of alpha, i.e., 5% in our study. The Bonferroni correction falls under the subject of multiple significance tests. To quote the work of Bland and Altman:^49^ “Many published papers include large numbers of significance tests. These may be difficult to interpret because if we go on testing long enough, we will inevitably find something that is significant. We must beware of attaching too much importance to a lone significant result among a mass of nonsignificant ones.”

In the current context, there are two significance tests, the first for the mAs effect (with 6 levels) and the second for the reconstruction effect (with 2 levels). For the mAs-effect, the DBMH procedure accounts for the six pairings and maintains the NH rejection rate at 5%. In other words, if the study were repeated 2000 times independently under NH true conditions (obviously this is only possible using a data simulator), there would be about 100 incorrect rejections of the NH for the mAs-effect. However, one is also attempting to draw conclusions about the effect of the two reconstruction algorithms, i.e., applying a second significance testing procedure. Again, the DBMH procedure maintains the Type I error rate at 5% for this comparison, so for 2000 simulations, one expects about 100 incorrect NH rejections for the reconstruction effect. The question arises to what extent the set of specific simulations where the NH was rejected for the mAs comparison (e.g., the 23rd, 30th, …, 1940th simulations, for a total of about 100) are common or distinct from the set of specific simulations that incorrectly rejected the algorithm effect. If the two sets are identical, then one still has a total of 100 NH rejections and the overall NH rejection rate is 5% and no correction is needed, which is the best-case scenario. The worst-case scenario is that the two sets are completely different, in which case the total number of NH rejections is 200. The only way to control this is to set a more stringent criterion for rejecting the NH. For example, if the criterion were set to reject 2.5% of the time for each type of comparison, there would be 50 NH rejections for the mAs comparison study and 50 different rejections for the algorithm comparison study, for a total of 100 NH rejections. To summarize, the Bonferroni correction involves using a smaller value of alpha, equal to the desired value divided by the number of significance tests. It is a conservative correction that, depending on the correlations between the two significance test results, tends to yield an effective alpha of less than 5%. A conservative correction is not always desirable because it leads to loss of statistical power and more sophisticated procedures are available.^50^

## APPENDIX B: JAFROC STATISTICS AND DEGREES OF FREEDOM

jafroc software reports the results of an overall *F*-test of the NH that all modalities being tested have identical FOMs. The analysis obtains two estimates of variance, the first due to the differences between modalities and the second due to other causes. If the observed ratio of the first variance to the second variance is large enough, the FOMs are expected to be significantly different. The ratio follows the *F*-distribution, which is characterized by two quantities called the numerator and denominator degrees of freedom, *ndf* and *ddf*, respectively. In general, if the ratio of the two variances is large and the degrees of freedom are large, the study tends to be more significant (smaller *p*-value).^51^
